# Phenotypic and Functional Diversity in Regulatory T Cells

**DOI:** 10.3389/fcell.2021.715901

**Published:** 2021-09-23

**Authors:** Louisa E. Sjaastad, David L. Owen, Sean I. Tracy, Michael A. Farrar

**Affiliations:** ^1^Department of Laboratory Medicine and Pathology, Center for Immunology, Masonic Cancer Center, University of Minnesota, Minneapolis, MN, United States; ^2^Division of Hematology, Oncology and Transplantation, Department of Medicine, University of Minnesota, Minneapolis, MN, United States

**Keywords:** regulatory T cells, autoimmunity, differentiation, fat, brain, skin, muscle

## Abstract

The concept that a subset of T cells exists that specifically suppresses immune responses was originally proposed over 50 years ago. It then took the next 30 years to solidify the concept of regulatory T cells (Tregs) into the paradigm we understand today – namely a subset of CD4+ FOXP3+ T-cells that are critical for controlling immune responses to self and commensal or environmental antigens that also play key roles in promoting tissue homeostasis and repair. Expression of the transcription factor FOXP3 is a defining feature of Tregs, while the cytokine IL2 is necessary for robust Treg development and function. While our initial conception of Tregs was as a monomorphic lineage required to suppress all types of immune responses, recent work has demonstrated extensive phenotypic and functional diversity within the Treg population. In this review we address the ontogeny, phenotype, and function of the large number of distinct effector Treg subsets that have been defined over the last 15 years.

## Thymic and Peripheral Tregs

Tregs develop via two different pathways: differentiation in the thymus from CD4+ thymocytes, or conversion of appropriately stimulated naïve CD4+ T cells in peripheral organs (Josefowicz et al., 2012a). Thymus-derived Tregs (tTregs) typically arise from self-reactive thymocytes. Thymocytes that interact more strongly with self-antigens differentiate into Treg progenitors characterized by increased expression of the TNF receptor superfamily members GITR, OX40 and TNFR2. Increased expression of these TNFR superfamily members renders these Treg progenitors more sensitive to IL-2, a cytokine required for optimal differentiation of mature Tregs in the thymus (Mahmud et al., 2014). In the thymus, one can already see evidence of distinct Treg subsets. Specifically, thymocytes that receive stronger signals via their TCR preferentially differentiate into CD25+ FOXP3- Treg progenitors, while thymocytes that receive somewhat weaker signals preferentially differentiate into CD25-FOXP3^lo^ Treg progenitors. The development of these two distinct Treg progenitors involves distinct signaling pathways and give rise to mature Tregs that have distinct roles in preventing autoimmunity (Owen et al., 2019). Thus, even at the earliest stages of Treg development one can see evidence for Treg diversification.

Peripheral Tregs (pTregs) differentiate from naïve CD4+ T-cells following their egress from the thymus. As pTregs differentiate in response to recognition of non-self-antigens, they are abundant at mucosal surfaces, which are exposed to commensal microorganisms, food, and environmental antigens (Josefowicz et al., 2012b). Mice lacking pTregs develop inflammation and dysbiosis in the gut and lungs (Josefowicz et al., 2012b; Campbell et al., 2018), demonstrating the importance of this subset for maintaining tolerance at mucosal surfaces. TCR stimulation in the context of signaling by TGF-β and retinoic acids promote pTreg development (Coombes et al., 2007; Yadav et al., 2013). The cell surface receptor NRP1 and the transcription factor HELIOS have both been used to discriminate thymic-derived from peripherally induced Tregs (Thornton et al., 2010; Weiss et al., 2012; Yadav et al., 2012), but this is somewhat controversial (Akimova et al., 2011; Szurek et al., 2015) and may only be true under steady-state conditions in the absence of inflammation (Weiss et al., 2012; Yadav et al., 2012). Regardless of their initial origin, HELIOS-negative Tregs appear to be somewhat less stable with a greater propensity to secrete inflammatory cytokines (Bin Dhuban et al., 2015). Analysis of tTreg and pTreg TCR repertoires demonstrate minimal overlap suggesting that these subsets recognize distinct antigens (Lathrop et al., 2011; Yadav et al., 2013). Both peripheral and thymically induced Tregs have been shown to play complementary roles in preventing autoimmunity (Haribhai et al., 2011). This further extends the concept of specialized roles for distinct Treg subsets.

## Central and Effector Tregs

**FIGURE 1:**
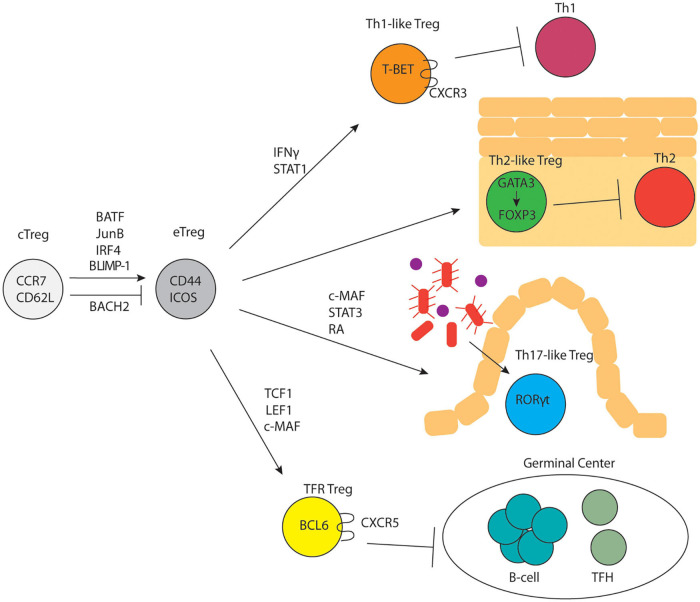
Treg subset differentiation. cTregs differentiate into eTregs in response to TCR signaling and upregulation of specific transcription factors. eTregs undergo differentiation into subsets that parallel CD4+ T-helper cell subsets and control distinct immune responses.

It is well known that conventional T cells undergo further differentiation in peripheral lymphoid organs upon antigen encounter and a variety of distinct effector T cell states have been characterized (Zhu et al., 2010). A similar concept was quickly established for Tregs as well. Tregs can be broadly divided into two subsets: central Tregs (cTregs) and effector Tregs (eTregs) based on their distinct anatomical locations and expression of activation-induced markers (Figure 1). cTregs are found primarily in secondary lymphoid organs and express high levels of the lymphoid homing markers, S1PR1, CD62L and CCR7 (Smigiel et al., 2014) and low levels of CD44, a marker of TCR activation. Additionally, cTregs are distinguished by high levels of the transcription factors, TCF1, SATB1, and BACH2 (Zemmour et al., 2018; Miragaia et al., 2019). Signaling through the IL-2R is essential for cTreg survival and maintenance (Malek et al., 2002; Fontenot et al., 2005; Burchill et al., 2007; Soper et al., 2007; Vang et al., 2008). Following TCR activation, cTregs can differentiate into eTregs; this process involves upregulation of activation induced markers, CD44, ICOS, PD-1, HELIOS, and 4-1BB (Miragaia et al., 2019), and concurrent downregulation of lymphoid homing proteins in favor of expression of chemokine receptors and adhesion molecules that enable Treg entry into, and accumulation in, non-lymphoid tissues. eTregs can be found in secondary lymphoid organs and exist in non-lymphoid tissues as tissue Tregs. In contrast to cTreg dependence on IL2, eTreg expression of ICOS is critical for their maintenance (Smigiel et al., 2014). Although TCR signaling is required for the differentiation of cTregs into eTregs (Levine et al., 2014), experiments using Nur77-GFP mice have demonstrated that cTregs express high levels of Nur77 similar to eTregs (Levine et al., 2014; Smigiel et al., 2014). This suggests that cTregs regularly encounter antigen but are precluded from conversion to eTregs. Two recent studies demonstrated that the transcription factor, BACH2, restrains the conversion of cTregs to eTregs by competing with IRF4 for DNA-binding sites and thereby repressing expression of genes involved in eTreg differentiation (Grant et al., 2020; Sidwell et al., 2020). The transcription factors IRF4, BATF, and JunB are expressed highly by eTregs and are critical for their differentiation and maintenance (Cretney et al., 2011; Delacher et al., 2017, 2020; Hayatsu et al., 2017; Koizumi et al., 2018). Specifically, mice with IRF4-deficient Tregs show multi-organ autoimmunity mediated by uncontrolled Th2 immune responses (Zheng et al., 2009). JunB facilitates the binding of IRF4 to a subset of its transcriptional target sites, like CTLA4 and ICOS, and Treg-specific deficiency in JunB results in multiorgan autoimmunity (Koizumi et al., 2018). BATF deficiency causes a reduction in tissue Tregs and their precursor effector Treg populations in the spleen (Delacher et al., 2017, 2020). ChIP-seq studies show that IRF4, BATF, and JunB colocalize near genes upregulated in eTregs, suggesting cooperative regulation of these genes (Koizumi et al., 2018). Another transcription factor, BLIMP-1, cooperates with IRF4 to establish the Treg effector program and promote expression of IL-10 in tissue Tregs (Cretney et al., 2011). In the absence of BLIMP-1, uncontrolled DNMT3A activity leads to increased methylation of the *Foxp3* locus and loss of suppressive activity (Garg et al., 2019). Single-cell ATAC- and single-cell RNA-sequencing of Tregs from lymphoid and non-lymphoid tissues has shown that Tregs undergo progressive, step-wise alterations in chromatin accessibility and gene expression to acquire a tissue Treg phenotype (DiSpirito et al., 2018; Miragaia et al., 2019; Delacher et al., 2020).

## Treg Subsets Parallel T Helper Cell Subsets

In 1986 Mossman and Coffman proposed that *in vitro* activated T helper cells could be driven to differentiate into two different subsets (Th1 versus Th2) characterized by distinct cytokine expression profiles; this was subsequently expanded to include Th9, Th17, and Tfh (and potentially other) subsets (Mosmann et al., 2005). Importantly, specific transcription factors including T-BET (Th1), GATA3 (Th2), RORγt (Th17), and BCL6 (Tfh) appeared to drive these distinct helper T developmental programs. Subsequently, multiple studies demonstrated that Tregs appeared to differentiate into parallel sets of cells (albeit with different functions from their effector counterparts), distinguished by the same sets of transcription factors (Figure 1). The plasticity of Tregs is controlled by cytokines, metabolism, and dietary factors. For example, NaCL has been shown to limit Treg suppressive function and drive them to adopt some Th17-like characteristics (Hernandez et al., 2015; Luo et al., 2019; Yang et al., 2020). The role of metabolic programming in shaping Treg functional plasticity was recently reviewed by Shi and Chi (2019) while the role of dietary factors involved in Treg function was recently reviewed by Arroyo Hornero et al. (2020).

### Th1 Tregs

Tregs expressing TBET were the first major Treg subset identified that appeared to parallel the T-helper differentiation paradigm. Koch et al. (2009) identified a population of TBET-expressing Tregs in the spleen that expanded during Th1-mediated inflammation (type I inflammation). Similar to Th1 cells, upregulation of TBET in Tregs is dependent on the IFNγ receptor and STAT1. TBET-expressing Tregs are found throughout lymphoid and non-lymphoid tissues and are a stable lineage that maintain TBET expression in perpetuity. TBET-deficient Tregs are unable to expand and control Th1-mediated inflammation due in part to their inability to upregulate the chemokine receptor, CXCR3 (Koch et al., 2009). In support of this idea, deletion of *Foxp3* in TBET-expressing cells or induced deletion of TBET-expressing Tregs leads to uncontrolled Th1 immune responses at steady state (Levine et al., 2017). These results indicated that TBET + Tregs play a critical role in controlling type I inflammation.

In Th1 cells, TBET promotes the expression of the effector cytokine, IFNγ, which is important for facilitating the type I immune response. Normally, TBET+ Tregs do not complete full Th1 differentiation and do not express IFNγ due to delayed expression of IL12Rβ (Koch et al., 2012). However, IFNγ-producing Tregs have been observed in several pathogenic settings. For example, during highly virulent infection with T. gondii, TBET+ Tregs acquired an effector phenotype and expressed IFNγ (Oldenhove et al., 2009). Tregs that produce IFNγ have been reported in inflammatory diseases including multiple sclerosis (Dominguez-Villar et al., 2011) and type I diabetes (McClymont et al., 2011). In a mouse model of melanoma, intratumoral Nrp1-deficient Tregs produced IFNγ, which enhanced anti-tumor immunity (Overacre-Delgoffe et al., 2017). Mechanisms involved in the development of Th1 Tregs and IFNγ-expressing Tregs were recently reviewed by Kitz and Dominguez-Villar (2017). The functional consequences of IFNγ production by Tregs are not well understood. However, production of Th1 cytokines by Tregs during inflammation is evidence of their phenotypic plasticity in response to environmental cues.

### Th2 Tregs

Similar to TBET-expressing Th1 Tregs, another population of Tregs was identified that expresses the canonical Th2 master transcription factor, GATA3. Although detectable in a number of lymphoid and non-lymphoid tissues, GATA3+ Tregs were initially found at barrier and mucosal surfaces including the skin, gut, and lungs (Wohlfert et al., 2011; Harrison et al., 2019). Initial studies demonstrated that mice with Treg-specific deficiency in IRF4, a transcription factor that is necessary for Th2 differentiation, developed uncontrolled Th2-mediated autoimmunity (Zheng et al., 2009). Subsequent studies determined that GATA3 expression is required for Treg stability and maintenance of *Foxp3* expression (Wang et al., 2011). Mice with Treg-specific deletion of GATA3 develop uncontrolled Th2 immune responses in the skin (Harrison et al., 2019). and a systemic inflammatory disorder later in life characterized by increased IFNγ, IL-4, and IL-17 production by effector T-cells (Wang et al., 2011). Thus, while TBET is required for suppressing Th1 responses, GATA3 appears to be more broadly important for Treg function. In support of this, *Foxp3* expression is reduced in GATA3-deficient Tregs (Wang et al., 2011). Co-immunoprecipitation experiments showed that FOXP3 and GATA3 interact in Tregs. Further, ChIP-qPCR demonstrated that FOXP3 binds to the *Gata3* locus and, conversely, GATA3 binds to the *Foxp3* locus (Rudra et al., 2012). This suggests that FOXP3 and GATA3 act as a complex to regulate gene targets and reciprocally regulate each other’s expression.

Recent single-cell RNA-seq experiments have demonstrated the importance of GATA3 in the differentiation of tissue Tregs at multiple sites (Delacher et al., 2017, 2020; Miragaia et al., 2019). GATA3-defined populations of non-lymphoid tissue Tregs are present in the skin, VAT, colon, lung, and liver. In the colon, the IL33R (ST2) is expressed primarily by GATA3-expressing Tregs (Schiering et al., 2014). GATA3 is also expressed in late tissue Treg precursor cells in the spleen. This population co-expresses several type 2 associated genes including *Il1rl1* (IL33R/ST2), *Areg*, *Il10*, and *Irf4*, indicating a role for Th2 programming in the differentiation of a subset of tissue Tregs.

Similar to IFNγ producing Th1 Tregs, there have been reports of pathogenic reprogramming of Th2 Tregs in food allergy (Noval Rivas et al., 2015). Mice bearing a gain-of-function *Il4ra* allele were sensitized and subsequently re-challenged with oral OVA, leading to severe anaphylaxis associated with the development of Tregs expressing IL-4, a Th2-associated cytokine driven by GATA3. In this model, an increase in GATA3+ Tregs was also observed upon allergen challenge. However, the authors did not directly address co-expression of IL-4 and GATA3. Deletion of IL-4 and IL-13 in Tregs protected mice from allergic anaphylaxis, indicating that Treg-produced IL-4 contributed to disease. Thus, while at steady state, Th2 Tregs do not generally produce Th2 cytokines, this has been observed under inflammatory conditions.

### Th17 Tregs

Tregs expressing the canonical Th17 transcription factor, RORγt, are highly prevalent in the gut and are important for controlling Th17-mediated immune responses. RORγt+ Tregs constitute approximately 15–30% of Tregs in the small intestine lamina propria and 40–80% in the colonic lamina propria (Sefik et al., 2015; Kim et al., 2016; Yang et al., 2016). RORγt+ Tregs are primarily pTregs induced by commensal microbiota (Kim et al., 2016). However, there is evidence that RORγt+ tTregs are also induced following immunization with the self-protein, myelin oligodendrocyte glycoprotein (MOG) (Kim et al., 2017). In the gut, retinoic acid and the transcription factor c-MAF are critical for the differentiation of RORγt+ Tregs (Ohnmacht et al., 2015; Wheaton et al., 2017; Xu et al., 2018). Initial evidence for the importance of RORγt+ Tregs in controlling Th17 responses came from mice with Treg-specific deficiency in STAT3, a transcription factor critical for expression of RORγt and Th17 differentiation; these mice developed uncontrolled Th17 responses and associated colitis (Chaudhry et al., 2009). In support of this, mice with Treg-specific deletion in RORγt were also more susceptible to colitis (Sefik et al., 2015). In contrast, a separate study demonstrated that mice with RORγt-deficient Tregs were unable to regulate Th2-mediated immune responses in the gut (Ohnmacht et al., 2015). The distinct phenotypes identified in mice with RORγt-deficient Tregs may be attributed to differences in microbiota composition and the anatomical location within the intestine from which cells were harvested and analyzed.

Despite co-opting Th17 features of Th17 programming, Tregs generally do not secrete Th17 cytokines at steady state. However, production of IL-17 by Tregs has been observed in mouse models of asthma and chronic arthritis, and in humans with psoriasis and Crohn’s disease (Bovenschen et al., 2011; Hovhannisyan et al., 2011; Komatsu et al., 2014; Sanchez Rodriguez et al., 2014; Massoud et al., 2016). Overexpression of the transcription factor, ID2, has been shown to promote Treg instability and expression of Th17 cytokines upon induction of experimental autoimmune encephalomyelitis (EAE), a mouse model of multiple sclerosis (Hwang et al., 2018). The tumor necrosis factor receptor super family (TNFRSF) members, CD27 and OX40, synergize to prevent aberrant expression of IL-17 in Tregs. Mice with CD27- and OX40-deficient Tregs were infected cutaneously with *C. albicans* to stimulate Th17-mediated skin inflammation. In this model, the double knockout Tregs expressed elevated levels RORγt and IL-17 suggestive of uncontrolled Th17-differentiation (Remedios et al., 2018). Thus, this study demonstrated that cell intrinsic expression of CD27 and OX40 by Tregs is required to limit Treg production of IL-17. Expression of the ST2/IL-33R on Tregs is also important for maintenance of Treg stability and resistance to acquisition of a Th17-like phenotype, including expression of RORγt and IL-17. Conversely, Treg expression of the IL-1R promotes instability and a Th17-like phenotype (Alvarez et al., 2019, 2020; Piccirillo, 2020). These studies demonstrated factors that influence Treg conversion to a Th17-like phenotype.

### T Follicular Regulatory Cells

T follicular regulatory (Tfr) cells are a subset of Tregs responsible for responding to and controlling the magnitude of germinal center (GC) reactions and subsequent antibody responses (Chung et al., 2011; Linterman et al., 2011; Wollenberg et al., 2011). Similar to T follicular helper (Tfh) cells, which promote GC responses and antibody production, Tfr express BCL6 and CXCR5, allowing them to localize to GCs. The transcription factors TCF1 and LEF1 are required for expression of BCL6 and subsequent Tfr development (Yang et al., 2019). Tfr also express high levels of ICOS, a costimulatory receptor, and PD-1, a coinhibitory receptor. ICOS knockout mice are deficient in Tfr while PD-1 knockout mice have increased Tfr with enhanced suppressive capacity (Sage et al., 2013).

The importance of Tfr in regulating GC responses was initially demonstrated by three separate studies. The first study demonstrated that mice with CXCR5-deficient Tregs, that were unable to enter the germinal center, had increased antigen-specific IgG compared to control mice in response to immunization (Wollenberg et al., 2011). Similar to this, the second study showed that mice with *BCL6-* deficient Tregs experienced higher antigen-specific antibody production in response to immunization (Chung et al., 2011). In contrast, the third report on Treg-depleted Foxp3-DTR mice and Tfr-deficient SAP KO:Foxp3 KO mixed bone marrow chimera mice showed that the absence of Tregs during germinal center reactions lead to decreased antigen specific GC B-cells and outgrowth of antigen non-specific B-cells (Linterman et al., 2011). To more definitively assess the role of Tfr in the germinal center reaction, mice were generated in which CXCR5 expressing Tregs could be inducibly deleted with diphtheria toxin. Using this model, the authors showed that Tfr function early, prior to GC formation, to limit GC B-cell development and antigen-specific antibody production in response to immunization. Tfr also regulated production of auto-antibodies that occur during immunization with foreign antigen and showed that Tfr are critical for controlling house dust mite-specific IgE during an allergic response (Clement et al., 2019).

Recent studies have revealed mechanisms by which Tfr regulate GC reactions and antibody responses. CTLA-4 mediates Tfr suppression of GC responses. Deletion of CTLA-4 on Tregs following their differentiation into Tfr leads to enhanced GC responses (Sage et al., 2014). Intriguingly, production of the neuropeptide, neuritin, by Tregs (likely Tfr) is important for controlling plasma cell differentiation from GC B-cells, limiting development of autoreactive GC-derived plasma cells, and limiting IgE responses (Gonzalez-Figueroa et al., 2021). These effects were attributed to neuritin’s effect on B-cells; however, it is possible that Treg derived neuritin is altering the activity of other cell types as well.

### Human Treg Subsets

While the majority of studies discussed above were done in mice, analogous subsets of Tregs have been identified in humans. Study of human Tregs and Treg subsets is complicated due to difficulty in accurate identification. A more detailed description of heterogeneity in human Tregs during health and disease can be found in recent reviews by Mohr et al. (2018) and Wing et al. (2019).

### Helper Treg Summary

Depending on the inflammatory setting, eTregs can undergo differentiation in parallel to effector CD4 T-cells and adopt limited Th programming. Expression of Th transcription factors allows Tregs functional plasticity to adapt to specific inflammatory environments and regulate the corresponding Th response. Several studies have shown functional deficits in Tregs that are unable to acquire Th programming. Although phenotypes associated with Th subtypes have been used to functionally categorize effector CD4 T-cells, a recent study has questioned this paradigm. Surprisingly, Kiner et al. (2021) has shown that effector CD4 T-cells are not distinguished by their respective Th classes when transcriptomes are analyzed by single-cell RNA-seq. Following Th-biased infections, effector T-cell transcriptomes did not form discrete clusters based on Th signatures. Partitioning of cells was most heavily dictated by the infection type not Th signature. Although this study did not address Treg heterogeneity, future studies should address whether a similar result would be observed for distinct subsets of Tregs responding to these infections.

## Tissue Tregs

In addition to Treg subsets defined by canonical Th transcription factors, there is extensive phenotypic and functional heterogeneity among Tregs that exist in different tissue types. Tissue Tregs are effector Tregs that have entered into and adapted to the specific tissue environment. There they serve to restrain immune responses, maintain tissue homeostasis, and promote tissue repair.

Recent use of single-cell RNA- and single-cell ATAC-seq to analyze Tregs from lymphoid and non-lymphoid tissues have elucidated the process by which circulating Tregs develop into tissue Tregs. Evidence suggests that Tregs acquire tissue Treg programming in a gradual, stepwise process that begins in the spleen and lymph nodes and is dependent on BATF (Hayatsu et al., 2017; Miragaia et al., 2019; Delacher et al., 2020). A Treg population found in the spleen that expresses low levels of the transcription factor, PPARγ, develops into tissue Tregs at multiple sites including skin, VAT, and liver demonstrating that tissue Treg precursors develop in the spleen prior to immigration to their respective tissues (Li et al., 2021a).

### Visceral Adipose Tissue Tregs

Early understanding of Tregs suggested their major function was to regulate immune homeostasis and immune response intensity. However, the observation of tissue Tregs in the steady-state challenged this assumption and supported a role outside more general immune homeostasis. First described in 2009, Tregs in the visceral adipose tissue (VAT Treg) represented the earliest example of a tissue-specific Treg subset whose function extends beyond the immune response (Feuerer et al., 2009). VAT Tregs remain one of the purest, and more well understood, examples of a Treg cell subset that adopts a phenotype and performs a function that is specific to their tissue of residency.

### Visceral Adipose Tissue Treg Derivation and Differentiation

The developmental scheme for VAT Tregs is well understood. Like most Tregs, the precursors for VAT Tregs also develop within the thymus (Kolodin et al., 2015). Interestingly, the VAT Treg compartment is reconstituted following Treg ablation in young but not old mice, suggesting that the perinatal thymus is critical for generating VAT Treg cell precursors. VAT Treg cell precursor development in the thymus occurs prior to 3 weeks of age as thymectomy in three-week old mice does not affect the accumulation of Tregs in the VAT (Kolodin et al., 2015). Several studies have suggested differences in thymic selection, and the function of Tregs, that develop in the perinatal versus adult thymus (Yang et al., 2015; Stadinski et al., 2019). However, why the adult mouse cannot replenish the VAT Treg cell compartment is unclear. Antigen presentation or antigen availability in the thymus of different aged mice could alter Treg cell output. This is supported by the observation that thymocytes expressing a transgenic TCR, isolated from VAT Tregs, are preferentially selected in the thymus during the first 2 weeks of life (Li et al., 2018). The endogenous targets of VAT related TCR’s have not been identified (Fernandes et al., 2020). However, if expression of the relevant antigen in the thymus is AIRE-dependent, the rapid reduction in AIRE+ mTEC following the perinatal period (Baran-Gale et al., 2020) could make VAT Treg cell selection inefficient in the adult thymus. Identification of the endogenous peptide that VAT Tregs recognize will facilitate a mechanistic understanding of VAT Treg cell selection in the thymus.

Despite developing in the thymus, VAT Treg cell differentiation is characterized as a two-step process that is initiated in secondary lymphoid organs. An important factor upregulated during VAT Treg differentiation is PPARγ, which is highly expressed in VAT Tregs and is critical for recruitment of Tregs to VAT (Cipolletta et al., 2012). However, it was initially unclear if PPARγ was upregulated by Tregs upon entering the VAT or upregulated in VAT Treg cell progenitors in secondary lymphoid organs (Cipolletta et al., 2012). The lack of reagents to easily detect PPARγ motivated the generation of *Pparg-Tomato* reporter mice to isolate and track cells that upregulate PPARγ. A small population of *Pparg-Tomato* positive Tregs can be identified in the spleen, although these Tregs expressed significantly less PPARγ than VAT Tregs (Li et al., 2018). Splenic PPARγlo Tregs do not express the majority of the VAT Treg cell signature but do exhibit an activated phenotype and upregulation of genes important for lipid metabolism. Transfer of PPARγlo Tregs led to more efficient seeding of the VAT compared to PPARγ- Tregs, despite being recovered at similar rates in the spleen, supporting the idea that PPARγlo Tregs are likely the progenitors for VAT Tregs (Li et al., 2018). However, a more recent study found that PPARγlo Tregs are the progenitors for Tregs in several non-lymphoid tissues other than VAT, including the liver and skin (Li et al., 2021a). It is unclear if Tregs seeding all tissues go through this PPARγlo intermediate. No matter the ubiquity of this PPARγlo intermediate in tissue Treg seeding, it clearly supports a two-step process for VAT Treg cell differentiation.

Several important factors required for the differentiation and/or retention of VAT Tregs have been described. A primary factor in VAT Treg cell development and VAT residency is TCR specificity. Though the target of VAT Treg TCRs has not be identified, TCR signaling is important at both steps of VAT Treg differentiation. Upregulation of PPARγ in VAT Treg precursors is significantly inhibited by MHC-II blockade (Li et al., 2018). Multiple observations also suggest VAT Tregs are responding to antigen within VAT. First, clonal expansions of VAT Treg TCRs are suggestive of local expansions that occur once VAT reactive Tregs enter the tissue (Feuerer et al., 2009; Kolodin et al., 2015). Second, Tregs that express a VAT Treg-enriched TCR transgene preferentially enter the VAT and undergo cell division more than their TCR transgenic negative counterparts. However, both populations adopt similar phenotypes within the VAT, a subset of which upregulate *Nr4a1* within the VAT confirming local recognition of VAT antigens (Li et al., 2018). Finally, VAT Tregs closely associate with MHC-II+ APCs in the VAT and MHC-II blockade antagonizes this association (Kolodin et al., 2015). Thus, TCR specificity and signaling are important for VAT Treg cell recruitment and retention. However, it remains unknown what antigen presenting cell (APC) population is required to present antigen in the secondary lymphoid organs and VAT to activate Tregs and what antigen they are presenting.

In addition to TCR signaling, several other factors are also important for VAT Treg cell differentiation. As discussed above, PPARγ is important for seeding the VAT by Tregs (Cipolletta et al., 2012) but is not important for the development of splenic VAT Treg cell precursors, as defined by KLRG1 and the IL-33 receptor, ST2 (Li et al., 2021a). While PPARγ expression is expendable for upregulation of ST2 in secondary lymphoid organs, PPARγ deficient Treg in the VAT do not properly upregulate ST2 (Kolodin et al., 2015). Sensing of IL-33 by ST2 is another important factor for VAT Treg cell phenotype and homeostasis. *Il1rl1* (ST2) knockout Treg have a significant defect in VAT accumulation and the adoption of the GATA3+ and KLRG1+ VAT Treg cell phenotype (Kolodin et al., 2015; Li et al., 2018). Treatment of mice with IL-33 expands the PPARγlo VAT Treg precursor in the spleen and drives expansion of the Treg compartment within the VAT (Kolodin et al., 2015; Li et al., 2018). Interestingly, provision of IL-33 expands VAT Tregs even while blocking TCR stimulation with MHC-II blockade (Kolodin et al., 2015), suggesting that IL-33 production alone can scale the size of the VAT Treg cell compartment. Nevertheless, TCR signaling may still be required for VAT Treg function in the context of IL-33 mediated expansion. Like other Tregs, VAT Tregs expand in response to IL-2 complex treatment (Feuerer et al., 2009). However, the expansion of VAT Tregs to IL-2 complex treatment is weaker than IL-33 treatment (Kolodin et al., 2015; Li et al., 2018) and unlike IL-33, IL-2 stimulation alone does not drive PPARγ upregulation (Li et al., 2018). PPARγ is not the only transcription factor that regulates VAT Treg phenotype. A recent study linked expression of the activated Treg transcription factor BLIMP1 to the VAT Treg phenotype, including expression of ST2 and KLRG1. Loss of BLIMP1 in Tregs reduced Treg production of IL-10 in VAT (Beppu et al., 2021). While coordination of PPARγ expression and IL-33 sensing stabilizes and scales the VAT Treg compartment, questions remain regarding the requirements for VAT Treg functionality.

### Visceral Adipose Tissue Treg Function

Visceral Adipose Tissue Tregs are critical regulators of organismal metabolic homeostasis and adapt to metabolic challenges. Visceral Adipose Tissue Tregs play an important role in regulation of glucose and insulin tolerance. Analysis of various mouse models of obesity revealed a strong negative association between VAT Treg proportions and insulin resistance (Feuerer et al., 2009). Depletion of Tregs in *Foxp3DTR* mice leads to increased insulin concentrations, and augmentation of VAT Tregs with IL-2 complex treatment bolsters insulin sensitivity and glucose clearance (Feuerer et al., 2009). These methods targeted all Tregs and not specifically the VAT Tregs. However, treatment of mice challenged with a high fat diet with the PPARγ agonist pioglitazone does not affect splenic Tregs but increases VAT Treg cell abundance and, coordinately, both glucose clearance and insulin sensitivity. This effect is VAT Treg cell specific as Treg specific knockout of PPARγ mitigated the response to pioglitazone (Cipolletta et al., 2012). However, VAT Tregs appear to reverse their role in metabolic homeostasis in the aged mouse. Depletion of VAT Tregs, via *Foxp3*^Cre^ mediated deletion of *Pparg*, rescued aging induced metabolic dysfunction (Bapat et al., 2015). The reason for these divergent observations in still unclear. However, deletion of PPARγ also reduced Tregs in subcutaneous adipose tissue (Bapat et al., 2015). The function of Tregs in non-VAT adipose depots is uncertain with some studies observing that Tregs promote metabolic homeostasis (Medrikova et al., 2015; Kalin et al., 2017; Fang et al., 2020) while others argue that Tregs promote metabolic disorder (Beppu et al., 2021). Regardless of the directionality, it is clear that adipose Tregs are a key regulator of systemic metabolic homeostasis.

While VAT Tregs are important in maintaining metabolic homeostasis, obesity leads to a diminished VAT Treg compartment. Mice treated with a high fat diet have diminished proportions of VAT Tregs and many of the remaining Treg have downregulated ST2 (Han et al., 2015). A high fat diet triggers increased production of pro-inflammatory cytokines, TNFα and IL-6, and macrophage infiltration in the VAT. Aberrant VAT inflammation can be rescued with IL-33 treatment, which correlated with VAT Treg cell expansion and recovery of ST2 expression (Han et al., 2015). The major producers of IL-33 in VAT are VAT mesenchymal stromal cells. Interestingly, a high fat diet initially decreases production of IL-33 by VAT mesenchymal stromal cells (VmSC), but following 4 months of high fat diet there is significantly more IL-33 producing VmSC in the VAT (Spallanzani et al., 2019). However, provision of IL-33 to mice lacking ST2 in Tregs leads to an increase in IL-33 producing VAT mesenchymal stromal cells (Spallanzani et al., 2019). Collectively, these results are suggestive of a feedback loop whereby VAT Tregs are both scaled by IL-33 but also regulate its production by VAT mesenchymal stromal cells. Importantly, increased production of IL-33 by VAT mesenchymal stromal cells is not sufficient to rescue VAT function in obesity (Spallanzani et al., 2019). However, expression of a soluble form of ST2 (sST2), which functions as a decoy receptor for IL-33, is induced in adipocytes following obesogenic challenge (Zhao et al., 2020). sST2 overproduction may explain the lack of Treg response to the elevated IL-33 produced during obesity. A recent study observed that plasmacytoid dendritic cells (pDC) progressively accumulate in VAT during obesity development (Li et al., 2021b). pDC production of IFNα reduces Treg expansion by inhibiting VAT Treg proliferation and increasing VAT Treg apoptosis (Li et al., 2021b). Several factors outside of homeostatic regulation of IL-33, perhaps increased inflammation or pro-inflammatory cytokines, or sST2 production, may regulate VAT Treg regulation during obesogenic challenges.

Female and male mice have profoundly different VAT Treg compartments and responses to metabolic challenges. A high fat diet induces an expansion of the VAT Treg cell compartment in females but reduces the VAT Treg compartment of males. Further, males on a high fat diet exhibit greater proinflammatory cytokine production and VAT macrophage infiltration than female mice (Pettersson et al., 2012). VAT Treg from female mice are phenotypically distinct from those in male mice, expressing a distinct transcriptome. Female mice have less VAT Tregs that express ST2, KLRG1, IL-10 and CCR2 than their counterparts in male mice (Vasanthakumar et al., 2020). In agreement with these phenotypes, IL-33 producing VAT mesenchymal stromal cells are more prominent in male mice and correlate with increased VAT Treg cell accumulation (Li et al., 2018; Spallanzani et al., 2019; Vasanthakumar et al., 2020). These effects are hormone dependent as androgen receptor deficiency in males and estrogen receptor-α deficiency in females reverse the VAT Treg phenotypes (Vasanthakumar et al., 2020). However, these hormones do not directly target Tregs but instead modulate IL-33 availability from the stromal cell compartment in the VAT (Vasanthakumar et al., 2020). These sex-specific characteristics lead to an improved metabolic response to obesogenic conditions in female mice (Vasanthakumar et al., 2020). An improved response to metabolic challenges appears to be important for healthy pregnancy as well. In agreement with the previous findings of sex hormones on the VAT Treg compartment, pregnancy induces a significant increase in VAT Tregs that is reversed upon *Foxn1-Cre* mediated deletion of RANK (Paolino et al., 2021). RANK deletion, presumably on thymic epithelial cells, caused a reduction in VAT Treg accumulation that antagonized insulin sensitivity in pregnant mice. This phenomenon led to the development of gestational diabetes and impairments in glucose homeostasis of mice gestated in mothers with reduced VAT Treg proportions (Paolino et al., 2021). Thus, sex hormone production, related to either sex or other physiologic processes, leads to strong differences in VAT Treg cell abundance and phenotype that causes differences in adaptation to metabolic challenges. In humans, females have a lower incidence and slower onset of type 2 diabetes than males (Kautzky-Willer et al., 2016), perhaps related to these differences in VAT and VAT Treg biology that have been observed in mice.

Visceral Adipose Tissue plays an important role in organismal metabolic homeostasis and VAT Tregs are crucial for VAT functionality. The metabolic perturbations caused by VAT Treg cell functional deficiency mimic those that contribute to increased incidence of type 2 or gestational diabetes and obesity. Bolstering the VAT Treg cell compartment represents an intriguing possibility to ameliorate these conditions. A recent report identified several mimotope ligands for a VAT Treg cell TCR, although the endogenous ligand remains unidentified. Interestingly, immunization of mice with this VAT Treg cell TCR ligand led to increased VAT Treg accumulation and improved insulin sensitivity in mice challenged with a high fat diet (Fernandes et al., 2020). This promising result suggests that specific targeting of VAT Tregs in humans may be beneficial for the treatment or prevention of metabolic disorders. However, despite the promise of targeting VAT Tregs in disease, the mechanism by which VAT Tregs function remains elusive. The most likely mechanism by which VAT Treg function is via control of macrophage infiltration and polarization in VAT, which precludes a VAT inflammatory environment and insulin resistance (Kolodin et al., 2015). More specific strategies to target VAT Tregs will facilitate the identification of important factors for VAT Treg regulation of VAT homeostasis.

### Muscle Tregs

Much like their Treg complements in VAT, muscle Tregs are a specialized population of Tregs that exhibit a unique TCR repertoire, transcriptome, and function. Despite being less well studied than VAT Tregs, the important mechanisms required for muscle Treg cell function are relatively well understood. Unlike their VAT Treg counterparts, the general function of muscle Tregs, largely in coordinating the tissue repair process by promoting tissue regeneration and reducing inflammation, is shared with other tissues as we will discuss in other areas of this review.

### Muscle Treg Derivation and Differentiation

Muscle Tregs, like VAT Tregs, appear to begin their development in the thymus. Muscle Tregs express *Nrp1* and *Helios* (*Ikzf2*), as well as a divergent TCR repertoire from Tconv found in the muscle (Burzyn et al., 2013). Muscle Tregs likely respond to self-antigen present in both the thymus and muscle tissue. In agreement with this, muscle Tregs contain an oligoclonal TCR repertoire that expands upon several types of muscle injury (Burzyn et al., 2013). Impressively, a single TCR clone, or subtle variation thereof, in injured muscle was shared between 11 mice, strongly supporting a dominant antigen driving expansion of the muscle Treg compartment (Burzyn et al., 2013). Further, TCR transgenic Tregs, expressing this publicly shared transgenic TCR, preferentially home to the muscle (Cho et al., 2019).

Like VAT Tregs, muscle Tregs appear to begin their differentiation in secondary lymphoid organs upon receiving TCR stimulation. Amphiregulin (AREG), a defining marker of the muscle Treg signature, is expressed in a small fraction of splenic Tregs that have a clonally expanded TCR repertoire which partially overlaps with the muscle Treg TCR repertoire (Burzyn et al., 2013). The overlap between the AREG+ population in the spleen and muscle strongly suggest an initial priming event in the secondary lymphoid organs that expands muscle specific Tregs. However, two phenomena cloud this interpretation. First, Tregs that express a muscle Treg cell specific transgenic TCR do not exhibit a strong TCR activation signature in the spleen but the TCR activation signature is upregulated when TCR transgenic Tregs arrive in the muscle (Cho et al., 2019). However, the lack of a strong TCR activation signature in the secondary lymphoid organs may just represent that a smaller number of muscle reactive Tregs are engaging cognate antigen in the secondary lymphoid organs versus in the muscle. Second, unlike VAT Tregs, muscle Tregs undergo retrograde trafficking from muscle to secondary lymphoid organs (Kuswanto et al., 2016). Thus, AREG+ splenic Tregs with an overlapping TCR repertoire with muscle Tregs may represent a retrograde trafficking event from fully differentiated muscle Tregs. However, given the ubiquitous activation signature in muscle Tregs (Burzyn et al., 2013), it is likely that their differentiation scheme does involve a priming event in secondary lymphoid organs before trafficking to the muscle where the muscle Treg cell signature is finalized (Cho et al., 2019). Supporting this, muscle Treg cell accumulation following injury is largely dependent on recruitment from secondary lymphoid organs (Kuswanto et al., 2016). TCR specificity is an important signal for muscle Treg cell development from conception in the thymus through initial activation in secondary lymphoid organs and finalization of muscle Treg development in the muscle.

Muscle Tregs share several factors with VAT Treg development although there are some exceptions. One such exception is the independence of muscle Treg accumulation on PPARγ and that PPARγ activation does not benefit the muscle Treg compartment (DiSpirito et al., 2018). It remains undetermined if muscle Treg progenitors go through the same PPARγlo intermediates as other tissue Treg subsets. However, given that PPARγlo tissue Treg precursors are not dependent on PPARγ, this remains a possibility. Nonetheless, like VAT Tregs, muscle Tregs express ST2 (Burzyn et al., 2013) and IL-33 is an important factor for their accumulation and response to injury (Kuswanto et al., 2016). IL-33 availability scales the size of the muscle Treg compartment and is upregulated in both mice and humans in response to muscle damage (Kuswanto et al., 2016). IL-33 is induced upon muscle injury, driving the expansion of muscle Tregs to facilitate muscle repair (Kuswanto et al., 2016). Interestingly, older mice, greater than 6 months of age, have reduced IL-33, both at steady-state and in response to injury (Kuswanto et al., 2016). Mesenchymal stromal cells in the muscle are the primary producers of IL-33 and injury induces their production of IL-33 within hours after injury (Kuswanto et al., 2016). Muscle mesenchymal stromal cells congregate with sensory neurons in the muscle and express a number of neuropeptides and neuropeptide receptors including calcitonin gene-related peptide (CGRP) (Wang et al., 2020). Treatment of mice with CGRP drives muscle IL-33 production and muscle Treg cell accumulation (Wang et al., 2020), suggesting that pain sensing after injury helps bolster the repair program. Upon muscle injury, sensory neurons and muscle mesenchymal stromal cells coordinate to increase IL-33 availability to recruit and expand muscle Tregs to facilitate proper muscle repair and regeneration.

### Muscle Treg Function

Muscle Tregs function broadly to promote muscle repair via immune dependent and independent mechanisms. Following injury, muscle Tregs expand peaking at 4 days post-injury and remaining elevated at least 1-month post-injury (Burzyn et al., 2013). Treg cell depletion impairs muscle regeneration due to increased muscle fibrosis and decreased satellite cell differentiation and increases muscle damage markers in a mouse model of muscular dystrophy (Burzyn et al., 2013). Conversely, bolstering muscle Tregs, either via IL-2 complex (Burzyn et al., 2013) or IL-33 (Kuswanto et al., 2016) treatment, improves muscle damage markers and facilitates superior muscle regeneration. Finally, as discussed above, aged muscle contains fewer Tregs due to reduced IL-33 availability, resulting in a fibrotic response to muscle injury. However, treatment of aged mice with IL-33, which rescues muscle Treg cell proportions, allows for partial restoration of muscle regeneration (Kuswanto et al., 2016). Both gain and loss of function experiments, as well as correlative observations in aged mice, confirm the importance of muscle Tregs in mediating the regeneration of functional muscle tissue following injury.

Muscle injury stimulates an immune response characterized by recruitment of macrophages, peaking at day two post-injury, followed by NK cells, CD4+ and CD8+ T cells by day four (Panduro et al., 2018). Infiltrating macrophages are comprised of two broad subsets – MHCII- and MHCII+. Treg depletion causes an expansion of MHC-II+ macrophages in the muscle along with increased intramuscular IFNγ production from NK cells, CD4+ and CD8+ T cells (Panduro et al., 2018). MHC-II expression on macrophages is important for IFNγ production by NK cells and CD4+ T cells; however, Treg cell expansion is independent of macrophage MHC-II (Panduro et al., 2018). Treatment of mice with recombinant IFNγ partially phenocopies the excessive inflammation and increased fibrosis observed in Treg cell depleted mice (Panduro et al., 2018). Early pro-inflammatory immune responses following injury are necessary to remove debris and damaged cells. However, this response must be shifted later to promote appropriate repair and regeneration. This latter phase of the injury response is promoted by Treg cell expansion and anti-inflammatory macrophage polarization (Panduro et al., 2018). All the mechanisms that Tregs employ to promote this response are unknown but regulation of macrophage polarization and IFNγ production are crucial to muscle regeneration.

Compromised intramuscular Treg cell function also causes immune-independent defects in muscle regeneration and repair. During muscle injury, muscle progenitor cells, termed satellite cells, differentiate into myoblasts and eventually a functional myotube to regenerate functional muscle tissue (Tidball, 2011). Treg cell depletion reduces the capacity of satellite cells to undergo myogenic differentiation (Burzyn et al., 2013). A substantial fraction of muscle Tregs express AREG (Burzyn et al., 2013), the epidermal growth factor receptor ligand critical for tissue repair (Berasain and Avila, 2014). Amphiregulin treatment rescues the myogenic potential of satellite cells and facilitates muscle regeneration during Treg cell depletion (Burzyn et al., 2013). This suggests that AREG production by Tregs is critical for muscle repair and regeneration via immune-independent effects on myogenesis. Indeed, Treg specific-deletion of *Areg* causes defective repair in the lung following influenza induced pathology, another model of inflammatory regeneration (Arpaia et al., 2015). Treg derived AREG has been linked to repair in other tissues including the CNS (Ito et al., 2019) and skin (Nosbaum et al., 2016). While some of the aspects of muscle Treg cell function are generalizable to repair processes in other tissues, such as effector cell activation, macrophage polarization and AREG production, muscle Tregs do contain a unique and clonally expanded TCR repertoire. Further, there are transcriptional features specific to muscle Tregs compared to other identified Treg cell subsets (Munoz-Rojas and Mathis, 2021) – whether these genes confer tissue specific functionality to muscle Tregs remains an open question.

### Skin Tregs

The skin contains a high frequency of Tregs. In humans and mice Tregs make up 20–40% of CD4+ T-cells within the skin (Kalekar and Rosenblum, 2019). Tregs in the skin express a strong Th2-biased phenotype where approximately 80% of Tregs express GATA3 (Wohlfert et al., 2011; Harrison et al., 2019). Although GATA3+ Tregs are generally regarded to be of thymic origin, this is not entirely clear as pTregs are able to upregulate GATA3 to the same degree upon antigen exposure (Wohlfert et al., 2011). Deletion of GATA3 in Tregs unleashes Th2 immune responses by commensal microbe-specific T-cells (Harrison et al., 2019). Indeed, it has been shown that the skin is seeded early in life by Tregs that recognize and facilitate tolerance to commensal microbes (Scharschmidt et al., 2015, 2017). A separate study demonstrated the importance of Treg-specific GATA3 expression in control of dermal fibrosis induced by Th2 cytokines (Kalekar et al., 2019). Another study reported that, similar to GATA3, approximately 80% of Tregs from the skin express the transcription factor, RORa (Malhotra et al., 2018), which has a reported role in the development of ILC2s. This study did not report if these RORa+ Tregs co-expressed GATA3. Deletion of RORa in Tregs leads to heightened Th2-mediated allergic inflammation. Overall these reports demonstrate that skin Tregs are poised to restrain Th2-mediated responses. Although GATA3 and RORa have previously been shown to be associated with skin Tregs, new evidence from single-cell ATAC and single-cell RNA sequencing have shown that these transcription factors are expressed broadly in Tregs from many tissue types and their precursors in the lymphoid tissue (Delacher et al., 2017, 2020). By comparison to those in the blood, Tregs from human skin preferentially express the mitochondrial enzyme, arginase 2 (ARG2). Expression of ARG2 promotes accumulation of Tregs within the skin and adoption of a tissue Treg transcriptional signature. In psoriatic skin, ARG2 inhibits mTOR activity, which has been shown to negatively regulate Treg proliferation. *In vitro* experiments indicate that Treg ARG2 can metabolize extracellular arginine to inhibit effector T-cell proliferation (Lowe et al., 2019).

In addition to their immunosuppressive functions, skin Tregs expand following tissue damage and promote wound healing and tissue repair. The majority of skin Tregs express ST2 (Delacher et al., 2020), the receptor for IL-33 that is released by cells in response to stress and damage. In Tregs, signaling through ST2 leads to the production of AREG, a protein with documented tissue repair function (Arpaia et al., 2015). In support of this, a recent study showed that Tregs in the skin expand following UVB irradiation and express AREG and the endogenous opioid precursor, PENK (Shime et al., 2020). Similar to AREG, PENK has been shown to promote wound healing and tissue repair (Shime et al., 2020). Tregs from wounded skin also express the AREG receptor, EGFR. Deficiency of EGFR in Tregs led to decreased accumulation of Tregs in the wounded skin and reduced wound closure suggesting a potential autocrine role of AREG in Tregs during tissue repair (Nosbaum et al., 2016). In response to acute epithelial injury of the skin, Tregs promote barrier repair by inhibiting the activity of Th17 cells and neutrophils (Mathur et al., 2019). Related to their role in tissue repair, skin Tregs have ascribed importance in facilitating hair follicle stem cell proliferation and differentiation through their expression of Jagged-1 (Ali et al., 2017). More information on the role of Tregs in skin injury can be found in a review by Boothby et al. (2020).

### Intestinal Tregs

Intestinal Tregs are important for facilitating tolerance to commensal microbes and environmental antigens. The intestine is regularly exposed to environmental antigens making it an ideal location for the induction of pTregs. Studies attempting to identify the origins of Tregs in the gut were conflicting. Analysis of TCR hybridomas bearing TCRs from murine colonic Tregs demonstrated their reactivity to colonic bacterial isolates. T-cells with retrogenic TCRs from colonic Tregs converted into Tregs in the colon but were unable to undergo diversion to the Treg lineage in the thymus (Lathrop et al., 2011). This suggested that colonic Tregs were pTregs rather than tTregs. However, a conflicting report showed that most colonic Tregs shared TCRs with thymic Tregs indicating a likely thymic origin (Cebula et al., 2013). Both studies agree that TCRs from colonic Tregs recognize microbial antigens.

While the origin of intestinal Tregs is debated, they seem to be composed of at least three populations of Tregs: RORγt+ pTregs, RORγt- pTregs, and GATA3+ tTregs. As mentioned previously, RORγt+ pTregs dominate the Treg population in the colon, while in the small intestine a substantial portion of pTregs do not express RORγt and are dependent on the presence of dietary antigens (Kim et al., 2016). Recent evidence demonstrates that the frequency of RORγt+ Tregs in the colon is stably transmitted from the mother to the progeny. This phenomena is dependent on levels of maternally transferred IgA which coat intestinal microbes and inhibit RORγt+ Treg induction (Ramanan et al., 2020). The enteric nervous system has also been shown to influence intestinal Tregs. Mice deficient in the neuropeptide precursor, TAC1, had increased frequencies of RORγt-expressing Tregs in the colon. Conversely, mice fed capsaicin, which stimulates neurons and release of neuropeptides, had decreased frequencies of RORγt Tregs (Yissachar et al., 2017). Stimulation of enteric neurons by microbes lead to release of neuronal IL-6 which promoted induction of RORγt+ Tregs (Yan et al., 2021). Tregs in the gut express high levels of the immunosuppressive cytokine, IL-10 (Atarashi et al., 2011). In germ free mice, the frequency of IL-10 producing Tregs in the colon, but not the small intestine, is significantly reduced indicating that these are likely pTregs (Atarashi et al., 2011; Ohnmacht et al., 2015). Recent studies have shown that Treg produced IL-10 plays an important role in promoting intestinal stem cell renewal (Biton et al., 2018). A distinct population of Tregs in the colon express high levels of GATA3 and ST2 (Wang et al., 2011; Wohlfert et al., 2011; Schiering et al., 2014). *In vivo* treatment with IL-33 expanded ST2+ colonic Tregs. ST2-deficient Tregs were limited in their ability to accumulate in the colon during inflammation (Schiering et al., 2014). As ST2+ Tregs have associated tissue reparative functions due to their expression of AREG, it has been suggested that ST2+ Tregs in the gut are mediating repair processes during tissue damage.

Single-cell RNA-seq of murine colonic Tregs identified transcriptional heterogeneity that matched previous studies (Miragaia et al., 2019). Three subsets were identified: lymphoid tissue-like Tregs that expressed central Treg markers including *Ccr7, Sell*, and *Tcf7*, non-lymphoid tissue-like Tregs that expressed *Gata3* and *Areg* and correspond to the previously described GATA3+ thymic Treg population, and suppressive Tregs that expressed *Lag3, Il10, Gzmb, and Cxcr3* and resemble the known RORγt+ population. A portion of Tregs within the mesenteric lymph nodes have partially adapted the gene signature characteristic of the colonic Tregs indicating progressive acquisition of this tissue transcriptome from the draining lymph to node to the tissue. Additional information on the roles of intestinal Tregs can be found in reviews by Whibley et al. (2019) and Cosovanu and Neumann (2020).

### Tregs in the Central Nervous System

**FIGURE 2:**
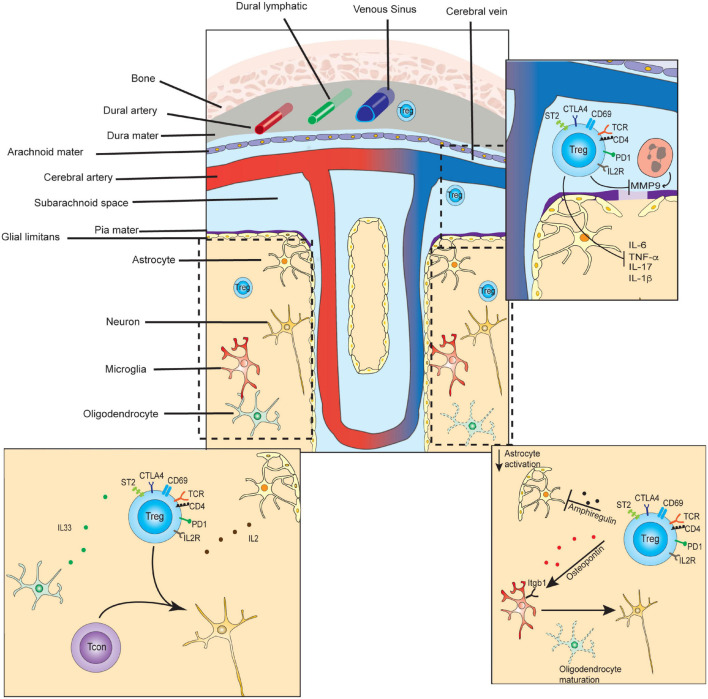
Select functions of Tregs in CNS tissue development, homeostasis, and repair. In the steady-state, small numbers of T-regulatory cells are found transiting the dura and leptomeninges, and persisting in the brain parenchyma. CD4+ T-cells of the parenchyma contribute to microglial maturation and synaptic pruning. (Lower left inset) In the steady state, parenchymal Tregs express markers of long-term residency and are maintained by IL-2 and IL-33, which may be primarily supplied by astrocytes and oligodendrocytes, respectively. (Top right inset) In the subacute or acute period following ischemic stroke, Tregs inhibit matrix metalloproteinase 9 (MMP9) secretion from neutrophils, preventing further breakdown of the blood-brain-barrier, and diminishing inflammation. (Bottom right inset) In the chronic period of ischemic stroke recovery, CNS Tregs suppress astrogliosis via the secretion of amphiregulin, and induce oligodendrocyte maturation via secretion of osteopontin.

Tregs infiltrating the CNS must navigate the unique compartmentalization and immune privilege of this anatomical space, which shapes their trafficking patterns, activation mechanisms, and tissue-specific functions. Recent investigations have revealed how immunological trafficking occurs between the brain, CSF, meninges, and periphery. The brain parenchyma and spinal cord are surrounded by cerebral spinal fluid (CSF), which is separated from systemic circulation (Figure 2). Specialized endothelial cells, surrounded by glial podocytes, form part of the pia mater meningeal layer, are bound by tight junctions, and compose the blood-brain barrier (BBB) (Daneman and Prat, 2015). The BBB, in turn, regulates bidirectional trafficking of cells and metabolic products between the CSF and the parenchyma. CSF ultimately drains into the dural sinus as well as into a separate series of specialized lymphatic vessels lining the dural sinus, before reaching the cervical lymph nodes (Louveau et al., 2015). Immune responses frequently begin in the meninges, which is a uniquely poised checkpoint to support or dampen inflammation in the CNS (Filiano et al., 2015). It is now increasingly understood that the interactions of Tregs with neuronal cells play critical roles in neurological development, neurodegenerative disease, and recovery from cerebrovascular infarction, as well as control of CNS-infiltrating cancers. Therefore, CNS Tregs likely represent a population that shares several properties with tissue Tregs of other spaces, but also harbor unique qualities reflecting specialized non-canonical functions.

In the steady state of healthy adult mice, approximately 2000 CD4 T-cells, including ∼150 Tregs, are found in the brain (Pasciuto et al., 2020). These are scattered at similar densities across most regions of healthy mouse brain, and are also found in similar proportions in rat cerebrum and biopsies of healthy human brain tissue (Xie et al., 2015). Higher frequencies, and persistent accumulation of Tregs, are seen following inflammation, and following cerebrovascular ischemia (Liesz et al., 2009b). They also increase in the tissues surrounding tumors involving the CNS, with a particularly high frequency of Tregs seen in carcinomas that have metastasized to the brain (Friebel et al., 2020). Compared to peripheral blood Tregs, steady-state brain Tregs are transcriptionally distinct, and express higher levels of activation markers (PD1, CTLA4, ICOS, KLRG1, CD103), and the residency markers ST2 and CD69 (Xie et al., 2015; Pasciuto et al., 2020). During severe inflammation in EAE models, CNS Tregs were also found to be transcriptionally dissimilar as compared to splenic Tregs (Garg et al., 2019). During the chronic phase of stroke recovery Tregs were found to express high levels of IL-10, KLRG1, ST2, and PPARγ, similar to VAT and muscle Tregs (Ito et al., 2019). However, CNS Tregs appeared to also specifically express genes involved in interactions with neural cells including neuropeptide Y (*Npy*), preproenkephalin (*Penk*), serotonin receptor type 7 (*Htr7*), and arginine vasopressin receptor (*Avpr1a*). Therefore, CNS Tregs are present in low frequencies in the steady state, but increase markedly during inflammation or following cerebrovascular insult, and adopt site-specific transcriptomes and immunophenotypes.

Several studies have now succeeded in delineating critical steps that Tregs undergo while trafficking between the periphery, the meninges, and the parenchyma of the brain. Parabiosis experiments demonstrated that immunophenotypically naïve Tregs only transiently enter the brain parenchyma, while activated Tregs experienced much longer dwell times (∼7 weeks) (Pasciuto et al., 2020). Furthermore, CNS-infiltrating Tregs converted to a CD69+ putative resident population at rates 100-fold higher than that of activated non-Treg CD4s (Pasciuto et al., 2020). Additional experiments found that TCR signaling was critical for both Treg entry and long-term persistence in the CNS. Specifically, usage of Nur77-GFP reporter mice revealed that, in contrast to CD69+ conventional T-cells, CD69+ CNS Tregs expressed high levels of the Nur77-GFP reporter. To formally test whether TCR engagement is required, Tregs bearing a TCR transgene reactive against the neuronal antigen MOG 35-55 (2D2) or the non-self-model antigen OVA (OT-II) were adoptively transferred into wild-type mice. Tregs expressing the 2D2 TCR transgene were enriched in the brain as compared to the periphery, and were preferentially enriched in the CD69+ population. In contrast, OT-II Tregs were undetectable in the same space. In separate work, OTII *Rag-/-* animals also showed no entry of Tregs into the brain during stroke recovery (Ito et al., 2019), although abundant numbers of peripheral endogenous Tregs entered the brain during the same time span. These more recent findings agree with earlier publications, which demonstrated that after the peak of EAE, effector T-cell populations contracted sharply, while regulatory populations were maintained at similar levels, implying a possible long-term resident regulatory population (Korn et al., 2007). Multiple observations therefore demonstrate that Tregs are able to migrate from the periphery to the brain and adopt a resident memory phenotype. This requires peripheral activation but further relies on continuous TCR engagement to retain Tregs and enable long-term residency.

Further studies have interrogated whether Tregs originate from central (thymus-derived) populations, or from peripheral induction. In both the steady state and at late-stroke recovery timepoints, the majority of brain Tregs express Helios, a putative marker of centrally derived Tregs (Ito et al., 2019). Classic EAE model experiments have also suggested a primary role for thymic-derived Tregs; when Foxp3/GFP- splenocytes were transferred from *Foxp3gfp* KI mice into *RAG-/-* recipients immunized with MOG, the transferred cells were absent from the CNS during peak inflammation, suggesting that peripheral conversion to regulatory status was not occurring (Korn et al., 2007). However some evidence supports the peripheral induction of Tregs in the CNS as well – for example, when OVA peptides were injected into the CSF of OT-II mice preferential accumulation of OT-II cells was observed in the meninges with ongoing polarization to Th1, Th17, or Treg phenotypes, followed by adoption of a resident memory phenotype (CD44+ CD69+) (Rustenhoven et al., 2021). Additionally, pseudotime analysis of T-cell populations in the CSF from patients with brain metastases suggested that naïve T-cells diverged into either Treg subsets or reactive/proliferating T cell states (Rubio-Perez et al., 2021). Conversion to Treg phenotypes in EAE models was previously shown to occur via cell-cell contact between activated T-cells and neurons in a TGFβ-dependent manner (Liu et al., 2006). Studies of TCR repertoire usage by CNS Tregs have revealed further insights into their origins as well as their antigen specificity; in both EAE and *T. gondii* infection models, analysis of TCR usage among regulatory and conventional T cells demonstrated a divergence in TCR repertoires between Tregs and Tcons, suggesting they do not extensively interconvert during peak inflammation (Nguyen et al., 2010; O’Brien et al., 2017). In post-stroke models, TCR repertoire usage of CNS Tregs is significantly limited as compared to splenic Tregs, and skewed toward usage of overlapping TCR clones (Ito et al., 2019), suggesting a distinct antigen-specific subset entered the CNS in this setting. Among patients with brain metastases, TCR analysis revealed that Treg populations had relatively high levels of unexpanded clones, and correspondingly low levels of proliferative marker expression, suggesting that *in situ* Treg expansion was minimal in this disease setting. Overall, the bulk of evidence so far suggests CNS Tregs may be either thymic-derived or peripherally induced and express distinct, relatively limited TCR repertoires, that reflect the influence of antigen binding.

Additional investigations have improved our understanding of the plasticity of CNS Tregs. At the peak of inflammation in EAE models, CNS Tregs were observed to upregulate signature genes of Th1 and Th17 cells but continued to express FOXP3 (Garg et al., 2019). This was due to constitutive expression of BLIMP1 in a STAT1-dependent manner, which antagonized IL-6-directed methylation of key regulatory regions of the *Foxp3* locus. BLIMP1 expression, in turn, has been shown to be maintained by stimulation of the tumor necrosis factor receptor 2 (TNFR2), which is highly expressed in CNS Tregs (Ronin et al., 2021). This is consistent with separate work demonstrating the importance of TNFRSF members at inducing and maintaining effector Tregs, in an NF-kB dependent manner (Vasanthakumar et al., 2017). FOXP3 expression can be downregulated under conditions of significant inflammation; Bailey-Bucktrout et al. (2013) demonstrated that MOG-specific Tregs, when transferred into recipient mice at the peak of EAE, lost FOXP3 expression, and gained effector T-cell properties including the capacity to secrete IFNγ. This was primarily observed in MOG-specific CNS Tregs rather than bulk polyclonal transferred Tregs. Among MOG-specific CNS Tregs, IL-2 complexes could block loss of FOXP3 expression upon transfer. Overall, this implies that CNS Treg identity is not necessarily fixed under severely inflammatory conditions, but influenced by signaling via the TCR, TNFRSF family members, and the availability of antigen and IL-2.

Tregs are known to depend on extrinsically provided IL-2 for maintenance and expansion in non-lymphoid tissues, including the CNS. Curiously, while conventional T-cells contract in the CNS following peak inflammation in EAE models, Tregs are maintained at similar numbers. This suggests they may derive IL-2 from alternative sources than the rapidly diminishing population of conventional T-cells. Astrocytes may serve this role, as they are known to constitutively express IL-2, and have been shown *in vitro* to function to maintain cerebral Tregs (Xie et al., 2015). IL-2 has been shown to be necessary for Treg expansion following stroke recovery (Ito et al., 2019) and its provision limits EAE-induced disease activity (Rouse et al., 2013; Ito et al., 2019). The therapeutic potential of this is not unrecognized – IL-2/IL-2c complexes, which selectively expand Treg populations, are protective in models of stroke and traumatic brain injury (Boyman et al., 2012; Gao et al., 2017; Zhang et al., 2018).

Maintenance of CNS Tregs is also influenced by IL-33 signaling, similar to other anatomical spaces. IL-33 is expressed widely throughout the healthy brain and is concentrated in white matter due to predominant expression in post-mitotic oligodendrocytes (Yasuoka et al., 2011; Jiang et al., 2012; Gadani et al., 2015; Fairlie-Clarke et al., 2018). ST2, the IL33 receptor, is highly expressed in brain Tregs at steady state. Conditional knockout of ST2 in FOXP3+ cells led to an exacerbation of EAE in mouse models, but did not affect overall cell numbers of CNS-infiltrating Tregs or T-effector cells (Hemmers et al., 2021). Instead, qualitative changes in Treg functionality were implied, reflected by their diminished capacity for suppression of IL-17A-secreting gamma-delta T-cells early in EAE development. ST2 KO CNS Tregs expressed lower levels of NF-kB pathway molecules, suggesting this signaling axis may be critical in CNS Treg maturation and functionality. While knockout of ST2 did not affect numbers of CNS Tregs in EAE models, diminished expansion was observed in ST2- or IL-33-deficient mice following middle cerebral artery occlusion (Ito et al., 2019). This may be explained by Treg-extrinsic effects of IL-33, as IL-33 provision leads to comparable increases in both ST2-sufficient and -deficient Tregs (Hemmers et al., 2021).

Specialized functions of CNS Tregs reflect their interactions with cells comprising the microglia, vasculature, lymphatics, neurons, and meninges. Tregs entering the CNS space have canonical anti-inflammatory roles, as well as contribute to tissue repair following a variety of immune- and non-immune-mediated insults. In mouse models using middle cerebral artery occlusion to induce permanent brain ischemia, multiple findings showed that Tregs affect tissue repair of the ischemic tissue, and ultimately impact neurological outcomes (Liesz et al., 2009b; Li et al., 2014, 2018). Similar findings support a role for Tregs in tissue repair during subacute/chronic phase after spinal injury (Raposo et al., 2014). Tregs accumulated in the infarcted tissue area several days after middle cerebral artery occlusion, and potentiated neurologic recovery (Ito et al., 2019; Shi et al., 2021). At day 14 after infarction, CNS Tregs had transcriptional signatures similar to other tissue Tregs including those present in VAT or injured muscle. However, they uniquely expressed the serotonin receptor type 7 gene, *Htr7*, among other genes. Neurologic recovery was deficient in the absence or blockade of serotonin activity, while serotonin potentiated neurologic restoration. The broad importance of serotonin to immune cell activity and autoimmunity has been reviewed elsewhere (Wan et al., 2020), but recent findings specifically identify a role for serotonin in modifying Treg functionality and frequency in the CNS at late-stroke timepoints (Ito et al., 2019). In middle cerebral artery occlusion models, Tregs contribute to neurologic recovery by suppressing astrogliosis via secretion of AREG. Additionally, Tregs induced oligodendrocyte maturation by secreting osteopontin as well as CCN3, a growth regulatory protein, thereby contributing to re-myelination (Dombrowski et al., 2017; Shi et al., 2021). In contrast to these later effects, other findings suggest Tregs markedly affect neurological recovery from ischemia and other insults during early timepoints as well. In the subacute/acute phase of ischemic stroke, meningeal Tregs inhibited the secretion of matrix metalloproteinase 9 from nearby neutrophils, resulting in better preservation of the blood-brain barrier (Li et al., 2013). This minimized cerebral inflammation and reduced infiltration of peripheral inflammatory cells into infarcted brain tissue. In the periphery, alterations of the frequency and function of Tregs in the spleen and blood are observed in patients following ischemic stroke, and in experimental models, implying that changes in Treg behavior in the periphery may also impinge on neurological outcomes (Offner et al., 2006; Liesz et al., 2009a; Hu et al., 2014; Ruhnau et al., 2016). Further studies are therefore needed to clarify the pleotropic roles that Tregs play in recovery from neurologic injury or ischemic recovery at multiple timepoints.

### Interferon Signature Tregs

Recent single-cell RNA-seq studies have identified a unique population of CD4+ conventional T-cells and Tregs that express strong interferon-stimulated gene (ISG) signatures. ISGs are a large family of protein coding genes that are expressed during viral infections and mediate anti-viral immunity. At steady state, a small subset of mature CD4SP thymocytes express a strong ISG signature (Hemmers et al., 2019). The purpose of these thymocytes remains unclear. Following infection with Salmonella or Citrobacter, a small population of effector CD4+ T-cells in the colon express high levels of ISGs (Kiner et al., 2021). Finally, during house dust mite challenge, a subset of lung CD4+ conventional T-cells expressed an ISG-signature (Tibbitt et al., 2019). CD4+ T-cell ISG expression was abrogated upon administration of IFNAR blocking antibody indicating that these cells are receiving signals from type I IFNs. In the skin draining brachial lymph node, a subset of Tregs express high levels of STAT1 and other ISGs (Miragaia et al., 2019). A similar population has been identified in the spleen, lungs, and gut (Lu et al., 2020). The function of these cells is also not known. It is possible that these conventional CD4+ T-cells and Tregs are drawn to an IFN-rich niche due to recognition of IFN-induced antigens and may play a role in tolerance to IFN-driven inflammation. In support of this possibility, one study has shown that Tregs are important for controlling IFNα and associated ISG production in a mouse model of psoriasis (Stockenhuber et al., 2018). Furthermore, a subset of house dust mite (HDM)-reactive CD4+ conventional T-cells and Tregs in humans express an ISG signature and these cells are more frequent in individuals without HDM allergy (Seumois et al., 2020), suggesting their preferential expansion in individuals without HDM allergy. This observation supports the hypothesis that Tregs bearing the ISG-signature could play a distinct immunosuppressive role during allergic responses. Further studies are needed to understand the function of these unique populations of CD4+ conventional T-cells and Tregs.

## Future Considerations

The studies discussed in this review have shed considerable light on the phenotype and function of several Treg subsets. However, further studies are required to understand the developmental timing and factors that drive tissue-specific Treg adaptations and how Treg phenotypes are altered during distinct immune responses. Further, the basic mechanism that licenses Treg activation and eTreg differentiation, required for all subsequent Treg specification, remain incompletely understood. Finally, as we discover more about Treg diversity, it will be important to develop novel tools that will enable precise interrogation of specific subsets and their functions *in vivo*. Currently, many studies make use of *Foxp3-DTR* mice to study Treg function in different contexts. While this is a powerful tool to study the consequences of Treg deletion, it does not allow for specific removal of distinct Treg subsets. Other groups have extensively utilized *Foxp3-CRE* mice crossed to mice with a floxed gene of interest to eliminate expression of that gene from Tregs. While this enables the study of that gene of interest in Tregs, it is possible that eliminating that gene may have broad effects on Treg biology and confound interpretation of functional changes that occur as a result. Finally, *Foxp3*-floxed mice crossed to mice expressing CRE driven by expression of a gene of interest have also been used regularly to eliminate particular Treg subsets. Use of this approach comes with the complication of potentially turning Tregs expressing the gene of interest into conventional CD4+ T-cells that could exert effector functions and preclude interpretation of the function of the subset of interest. While these murine models have provided great insight into Treg function, new tools are needed to isolate and manipulate particular Treg subsets *in vivo*.

The dynamics of Treg heterogeneity during immune perturbations have also been largely unexplored. For example, we do not know how proportions of Treg subsets within tissues change over the course of an infection or injury. scRNA-Seq based approaches should allow us to better characterize Treg subset dynamics during disease and enable better understanding of the function of distinct Treg subsets. Likewise, where tissue Tregs exert their function within a tissue, and which cells they are intimately interacting with remains an open area of exploration. Additionally, aside from expression of Thelper associated markers, there is limited information about how Treg subsets vary in the context of distinct infections and tissue damage or injury. Further studies using single-cell RNA-seq or related approaches should be informative in this context as they allow dissection of heterogeneity within the Treg population and allow identification of potentially novel Treg subsets.

Non-lymphoid tissue Tregs are diverse and express phenotypes that are distinct from those expressed by lymphoid tissues. While several recent single-cell RNA-seq experiments have characterized the transcriptional adaptation of lymphoid Tregs into non-lymphoid tissue Tregs, the factors that drive specific tissue adaptations and the timing of their expression are still not well understood. Thus, it will be important to understand when and how Tregs adapt to specific tissues and how they diverge phenotypically within one tissue.

## Author Contributions

LS, DO, and ST wrote the manuscript. MF edited the manuscript. All authors contributed to the article and approved the submitted version.

## Conflict of Interest

The authors declare that the research was conducted in the absence of any commercial or financial relationships that could be construed as a potential conflict of interest.

## Publisher’s Note

All claims expressed in this article are solely those of the authors and do not necessarily represent those of their affiliated organizations, or those of the publisher, the editors and the reviewers. Any product that may be evaluated in this article, or claim that may be made by its manufacturer, is not guaranteed or endorsed by the publisher.
